# A Comprehensive Review of Theaflavins: Physiological Activities, Synthesis Techniques, and Future Challenges

**DOI:** 10.1002/fsn3.70762

**Published:** 2025-08-06

**Authors:** Shaolong Du, Junyi Chang, Zhimei Zhou

**Affiliations:** ^1^ College of Chemical Engineering and Chemistry Hunan Normal University Changsha China; ^2^ Hunan Xinsheng Tea Science and Technology Co., Ltd. Shaoyang China

**Keywords:** challenge, health benefit, synthesis, theaflavin

## Abstract

Theaflavins (TFs), which are polyphenolic compounds characterized by a benzotropolone structure, serve as the primary quality and health‐promoting components in black tea. Recent investigations have disclosed various health advantages linked to TFs, especially their potential to act as lead compounds in the formulation of therapeutic drugs targeting severe acute respiratory syndrome coronavirus 2 (SARS‐CoV‐2), positioning them as a significant area of focus within food science and nutrition research. This review initially examines the primary physiological activities, mechanisms of action, and challenges related to TFs. It subsequently details the formation mechanism of enzyme‐catalyzed TFs from catechins. Building upon this groundwork, this review assesses the recent advancements in two in vitro synthesis methods of TFs: enzymatic oxidation and nonenzymatic synthesis. Finally, the challenges that arise during the large‐scale industrial implementation of these synthesis techniques are analyzed, and research strategies aimed at mitigating these issues are suggested. The primary goal of this review was to provide insightful perspectives and guidance for prospective research and industrial utilization of TFs.

## Graphical abstract

Theaflavins demonstrate significant pharmacological effects on various chronic diseases, while low availability and stability. In vitro synthesis methods, such as enzymatic oxidation and nonenzymatic synthesis, can significantly enhance the production of theaflavins, but industrialization still faces some challenges. Innovative technologies such as protein engineering, genetic engineering, and biomimetic catalysis are expected to be powerful tools for addressing the industrial challenges of theaflavins.

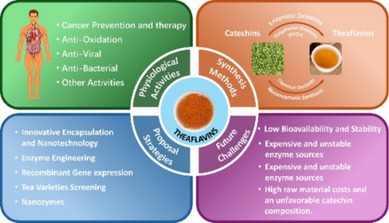

## 1 Introduction

**FIGURE 1:**
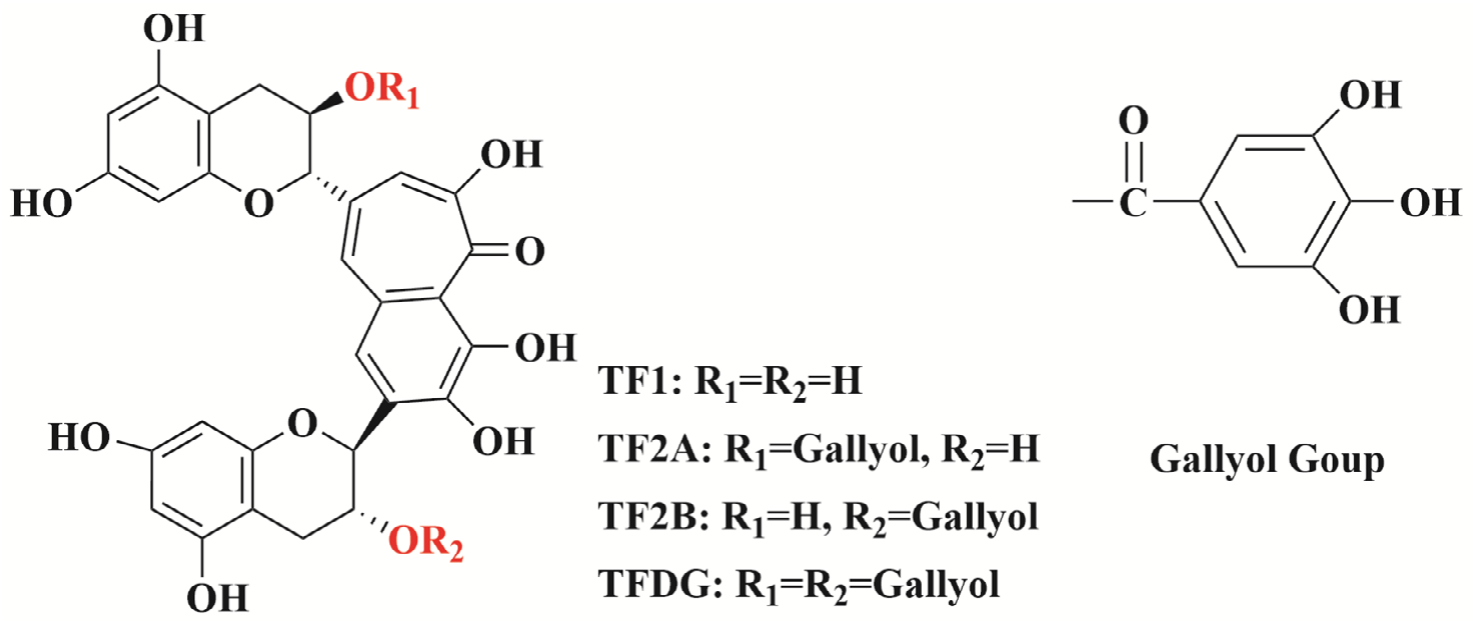
Chemical structural formulae of the four main theaflavins.

Theaflavins (TFs) are distinct golden‐yellow pigments that emerge during the fermentation process of black tea and are categorized as compounds with a unique benzotropolone structure (Sen et al. 2025). These pigments are synthesized through the enzymatic oxidative coupling of catechins, which are naturally occurring antioxidants found in tea. To date, researchers have identified over 20 distinct derivatives of theaflavin present in black tea. Among these derivatives, the four primary varieties include theaflavin (TF1), theaflavin‐3‐gallate (TF2A), theaflavin‐3′‐gallate (TF2B), and theaflavin‐3,3′‐digallate (TFDG) (Zhao et al. 2024; See Figure 1). Despite their relative scarcity, accounting for approximately 1% of the total solids in tea, these polyphenolic compounds play a crucial role in shaping the sensory characteristics of black tea infusions (Özdemir et al. 2028). Specifically, TFs significantly influence the brightness, strength, and freshness of the tea's flavor. Consequently, the content of TFs has become an essential biochemical marker for assessing the quality and classification of black tea. This evaluation is vital for determining the tea's market value, as higher concentrations of these compounds often correlate with superior sensory qualities and increased consumer preference.

Pharmacological investigations have demonstrated that TFs possess significant physiological effects, exhibiting potent antioxidant (Wang, Ren, et al. 2023), antiviral (Mhatre, Naik, and Patravale 2021), anticardiovascular (Sen et al. 2022), and antihyperglycemic (Li, Dong, et al. 2024) activities. Research indicates that TFs may exhibit enhanced physiological activity compared to (−)‐epigallocatechin gallate (EGCG), which is recognized as the most effective catechin (Ohgitani et al. 2021). However, the limited quantity of TFs present in black tea presents considerable challenges for their extraction, including low purity levels, high costs, and the presence of solvent residues, all of which hinder the rapid and effective expansion of the TFs industry (Dong et al. 2025). To overcome these challenges, various strategies for the in vitro synthesis of TFs have been developed, utilizing both chemical and enzymatic oxidation techniques (Liu et al. 2024). Although these methods still face issues such as low conversion ratios, the formation of complex by‐products, and difficulties in achieving high‐purity preparations, they provide valuable insights and experiences relevant to the large‐scale production of TFs (Takemoto and Takemoto 2018).

Recent reviews on the research of TFs have highlighted advancements in extraction and synthesis techniques (Zhao et al. 2025), interactions with gut microbiota (Dong et al. 2025), health benefits (Shan et al. 2024), and comprehensive metabolic pathways (Li, Li, et al. 2024). However, there has been limited attention on the industrial realization of TFs synthesis. The scarcity of low‐cost, high‐purity TFs and their derivatives has become a bottleneck that hinders the widespread application of these compounds. Therefore, this review will not only focus on the physiological activities of TFs but will also concentrate on the research progress and future challenges related to their industrial‐scale production. Specifically, this paper explores the latest advancements in the physiological activities of TFs, their formation mechanisms, and improvements in synthesis methods of TFs. Additionally, we analyzed the main challenges currently faced in the development of tea yellow pigments, such as low bioavailability and stability, limited human trials, and the lack of stable and efficient catalysts. Furthermore, we proposed strategies for future research in these areas. Our goal is to establish a theoretical foundation for ongoing research aimed at the efficient preparation, efficacy assessment, and product development of TFs, ultimately promoting the advancement of this promising research field.

## 2 Physiological Activities of TFs

**FIGURE 2:**
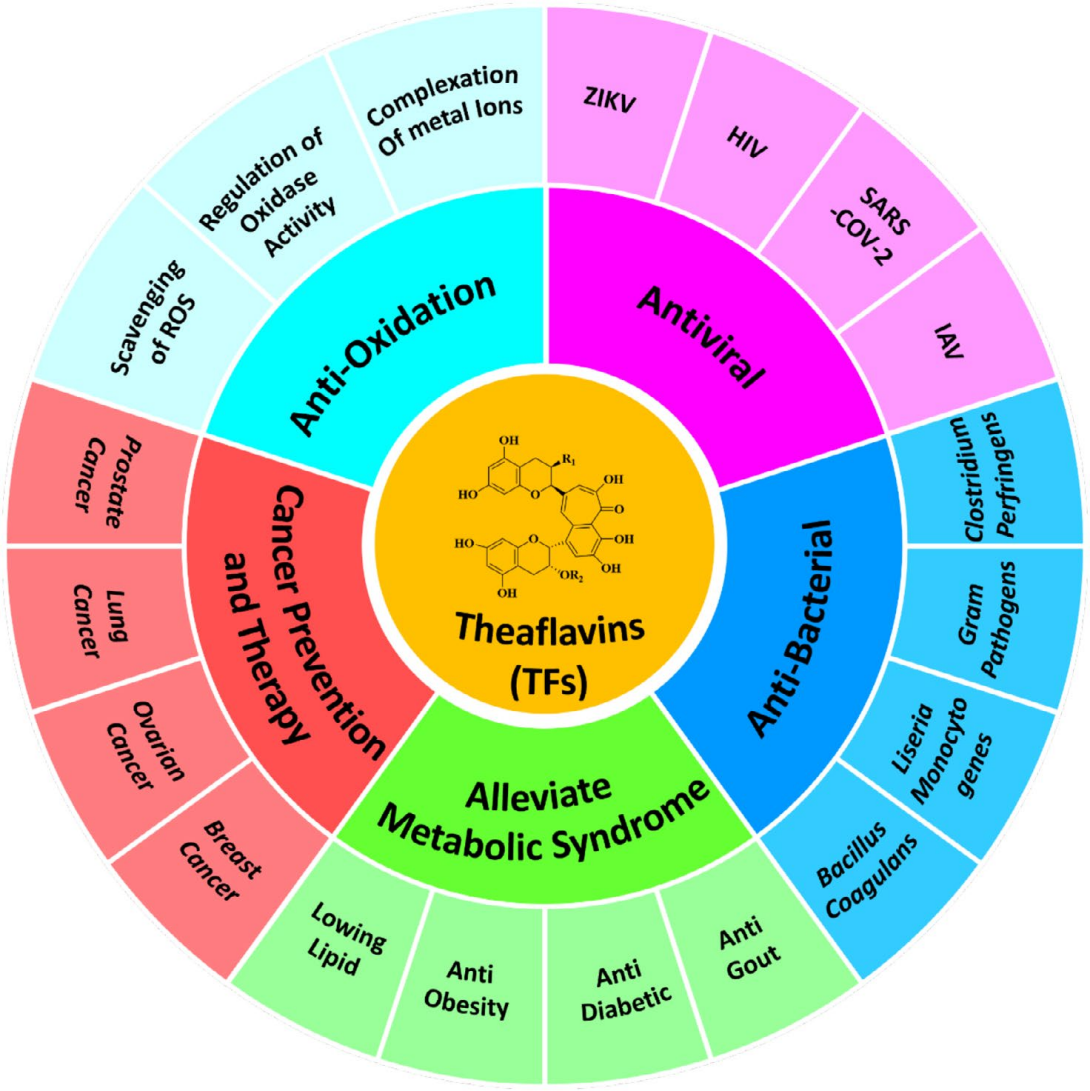
Physiology activities of theaflavins.

Theaflavins, as a primary quality and functional component of black tea, have attracted considerable attention due to their physiological activity (Liu et al. 2024). A multitude of in vitro and in vivo studies have demonstrated the broad‐spectrum physiological effects of TFs, which include antioxidant, anti‐inflammatory, anticancer, and antibacterial properties. Importantly, TFs have been shown to inhibit the SARS‐CoV‐2 virus, positioning them as one of the most promising candidates for the prevention and treatment of COVID‐19 (Mhatre, Srivastava, et al. 2021). Consequently, TFs present significant advantages in the prevention and treatment of lifestyle‐related and age‐related diseases, offering protective effects on various organs, including the skin, liver, kidneys, brain, and heart (see Figure 2).

### 2.1 Antioxidant and Free Radical Scavenging

A significant amount of research has demonstrated that black tea possesses significant antioxidant properties, primarily attributed to polyphenolic compounds such as catechins and theaflavins (TFs), which are identified as the primary antioxidant components in black tea (Mehrabi et al. 2025). In vitro experiments demonstrated that the antioxidant activities of the four major tannin fractions (TFs) were ranked as follows: TFDG>TF2A/TF2B>TF1. This ranking was established in comparison with the free radical scavenging activities of 2,2′‐Azinobis (3‐ethylbenzothiazoline‐6‐sulfonic acid; ABTS+), 1,1‐Diphenyl‐2‐picrylhydrazyl (DPPH), as well as the ferric‐reducing ability of plasma (FRAP) assays and total antioxidant capacity (Wang, Le, Wang, Yin, and Jiang 2023). Furthermore, it has been noted that TFDG displays superior antioxidant efficacy compared to epigallocatechin gallate (EGCG), the most potent antioxidant catechin. This enhanced ability is primarily associated with the varied phenolic hydroxyl structures present in TFs, which enable them to donate hydrogen atoms to interact with various free radicals, including superoxide anion radicals, hydroxyl radicals, and hydrogen peroxide. This interaction transforms these radicals into stable molecules and interrupts the chain reactions of free radicals (Vu et al. 2019).

Additional research has demonstrated that TFs are capable of binding to the active sites of oxidases, like xanthine oxidase and tyrosinase, which obstructs the release of catalytic products (Gothandam et al. 2019). This binding action hampers the catalytic function of these enzymes, lowers levels of reactive oxygen species (ROS), and alleviates oxidative damage (Sharma et al. 2020; Chandravadhana et al. 2020). Data obtained from electroanalytical studies focused on TFs and their interaction with copper ions indicated that TFs possess greater antioxidant capacity compared to EGCG (Chen et al. 2022). This increased effectiveness may stem from the unique fused ring structure and a higher count of hydroxyl groups present in TFs, facilitating their ability to chelate metal ions and form stable complexes, thus inhibiting the participation of metal ions in free radical formation. These results emphasize the diverse mechanisms through which TFs manifest their antioxidant properties, showcasing their promise as powerful natural antioxidants.

The exceptional antioxidant activity of TFs is attributed to their unique molecular structure, which combines a benzotropolone core with multiple hydroxyl groups (Sharma 2024). The benzotropolone structure serves as the fundamental basis for the antioxidant properties of TFs (Wang, Le, Wang, Yu, et al. 2023). On one hand, the conjugation effect within this structure disperses unpaired electrons on oxygen across the conjugated system, weakening hydrogen bonds and enhancing the reactivity of hydrogen atoms on the hydroxyl groups. This facilitates the donation of hydrogen atoms, enabling TFs to exert their antioxidant effects. Conversely, after donating hydrogen atoms, TFs are initially converted into phenoxy radicals, which subsequently undergo intramolecular electron transfer to form hydroxy cycloheptene radicals. These radicals exhibit the lowest reducing potential, further enhancing their antioxidant capacity (Kamal et al. 2025). The position and number of hydroxyl groups play a critical role in determining the antioxidant activity of TFs (Wang, Guo, et al. 2025; Wang, Zhao, et al. 2025). Specifically, the β‐pyrogallol and 3‐galloyl groups are key contributors, with the 3‐galloyl groups demonstrating stronger antioxidant activity than the β‐pyrogallol groups. Consequently, the antioxidant activity of TFs is positively correlated with the number of galloyl groups rather than their position (Sang et al. 2003). This structural insight underscores the importance of galloyl groups in enhancing the antioxidant potential of TFs, providing a molecular basis for their superior free radical scavenging and oxidative stress mitigation capabilities (Jiang et al. 2021).

### 2.2 Antiviral Activity

Viruses are parasitic infectious agents that rely on host cells to replicate their nucleic acids, demonstrating high virulence and transmissibility (Wang, Wang, et al. 2022). In recent years, recurrent viral disease outbreaks, such as coronavirus disease 2019 (COVID‐19), have posed significant threats to human life and health, highlighting the urgent need for effective antiviral strategies on a global scale (De et al. 2023; Li, Bai, et al. 2022; Li, Yuan, et al. 2022). However, the current repertoire of antiviral drugs remains limited. This challenge is exacerbated by rapid viral mutation rates and the emergence of drug‐resistant strains, leading to an increasing demand for novel “resistance‐proof” antiviral therapies (Chen et al. 2024; Paiva et al. 2022).

TFs have emerged as promising antiviral agents, exhibited broad‐spectrum antiviral activity while remaining nontoxic and free from side effects, which aligns with contemporary drug development priorities (Sima et al. 2023). Among the four primary TFs, TFDG has garnered particular interest due to its efficacy against a variety of viruses, including the novel coronavirus, influenza virus, Zika virus, and human immunodeficiency virus (HIV) (see Table 1). Current research is focused on elucidating the antiviral mechanisms of TFDG. In vitro cell cultures and animal models are utilized to assess viral nucleic acid replication, gene transcription, and protein expression through both qualitative and quantitative analyses, thereby shedding light on the molecular basis of the antiviral action of TFs (Deng et al. 2024).

**TABLE 1:** TFs and their derivatives with the potential of inhibiting the infection of viruses.

TFs source	Virus	Results	References
TF2B	Influenza viruses (H1N1‐UI182, H1N1‐PR8, H3N2, H5N1)	TF2B inhibits the replication and proliferation of influenza virus TF2B enhances the survive rate of mice infected with H1N1‐UI182 TF2B can the inflammatory cytokines triggered by the influenza virus through the modulation of the TLR4/MAPK/p38 signaling pathway	Sima et al. (2023)
TF1, TF2B	Zika virus (ZIKV)	TF1 and TF2B markedly reduced the replication of the ZIKV/Z16006 toxic strain in BHK and Vero cells by obstructing both the replication and release of ZIKV TF2B enhances the survival rate of mice infected with ZIKV The treatment with TF2B notably diminished the levels of cytokines (such as IL‐6, IL‐1β, and TNF‐α) along with chemokines (including CCL2, CCL5, and CXCL10) that were triggered by ZIKV infection	Deng et al. (2024)
Theaflavin gallate	Zika virus (ZIKV)	Theaflavin gallate demonstrated a strong inhibitory effect against Zika virus (ZIKV) protease, with an inhibitory constant (Ki) value of 0.40 μM	Coelho et al. (2022)
TFDG (ZP10)	Zika virus (ZIKV)	ZP10 (TFDG) has been recognized as an effective inhibitor of the Zika virus NS2B‐NS3 protease (ZIKVpro), with an IC_50_ value of 2.3 μM ZP10 demonstrates a dose‐dependent effect on the inhibition of ZIKV replication, with an EC_50_ value of 7.65 μM ZP10 interferes with the cleavage of the viral polyprotein precursor in cells infected with ZIKV, as well as in those expressing the minimally self‐cleaving NS2B‐3 protease, which ultimately led to reduced viral growth	Cui et al. (2020)
Theaflavin‐concentrated tea extract (TY‐1)	Influenza A virus (IAV)	TY‐1 demonstrated both concentration and time‐dependent virucidal effects against IAV TY‐1 demonstrates various pathways for inactivating IAV, which includes decreasing the intensity of the hemagglutinin band, causing the formation of extra high molecular weight bands or ladders, lowering the intensity of the neuraminidase (NA) band, and diminishing both hemagglutination and NA activities	Mohamed et al. (2022)
TFDG	SARS‐CoV‐2	TFDG was chosen as a therapeutic option for SARS‐CoV‐2 because it can undergo favorable conformational changes during binding and maintains a strong affinity for the binding site of the main protease	Abraham Peele et al. (2020)
TFs	African swine fever virus (ASFV)	TFs can effectively inhibit the replication of African swine fever virus (ASFV) in porcine primary alveolar macrophages (PAMs) TFs suppress ASFV replication by enhancing the AMPK signaling pathway TFs inhibit ASFV replication by reducing the intracellular levels of total cholesterol and triglycerides in cells infected with ASFV	Chen, Wang, et al. (2023), Chen, Wei, et al. (2023)
TFs	Lumpy skin disease virus (LSDV)	TFs inhibit LSDV replication, with an IC_50_ of 10.05 μM TFs obstruct LSDV by inhibiting viral entry into cells and its subsequent replication stages, with IC_50_ value of 11.5 μM	Wang et al. (2024)
TFDG	SARS‐CoV‐2	TFDG emerged as one of the highest‐ranking compounds after three independent virtual screenings of 598,435 compounds	Gan et al. (2024)
TFDG	SARS‐CoV‐2 (Wuhan and Omicron strains)	Administration of 1 mM TFDG for 10 min reduced the infectivity of both the Wuhan and Omicron strains to about 0.05% and 3%, respectively	Manish et al. (2022)

Currently, the specific mechanism by which theaflavins exhibit antiviral properties remains unclear. However, some studies suggest that theaflavins possess a unique structure characterized by a benzophenone backbone and polyhydroxyl groups. Furthermore, there appears to be a significant interaction between theaflavins and the main protease of the virus, which may influence various stages of viral invasion within the host organism (Verma et al. 2023; Jain et al. 2022).

During the stages of viral adsorption and infection of host cells, TFs can inhibit the adhesion and internalization of viruses by interacting with viral surface proteins or host cell receptors. The SARS‐CoV‐2 virus enters host cells through the specific binding of its spike glycoprotein (S protein) to the host cell surface receptor angiotensin‐converting enzyme II (ACE2). Blocking the interaction between the S protein and ACE2 may prevent the invasion of this virus (Qin et al. 2023). Studies have demonstrated that TF2A forms stable hydrogen bonds with the receptor binding domain (RBD) of the SARS‐CoV‐2 S protein, thereby obstructing the interaction between the S protein and ACE2. This significantly reduces the virus's adhesion to respiratory epithelial cells and consequently inhibits the initial infection process (Lu, Lung, et al. 2024).

During the replication and assembly stages of the virus, TFs may interfere with the key enzymes required for viral nucleic acid replication (Gogoi et al. 2021). Research has demonstrated that TF1 can upregulate the 5′‐AMP‐activated protein kinase (AMPK) signaling pathway in both African swine fever virus (ASFV)‐infected and uninfected cells, disrupting lipid metabolism and inhibiting ASFV proliferation in a dose‐dependent manner (Lung et al. 2020). TFDG (ZP10) has been shown to directly bind to the NS2B‐NS3 protease (ZIKVpro) of the Zika virus (ZIKV), and docking models further reveal that ZP10 can interact with several key residues within the proteolytic cavity of ZIKVpro, thereby inhibiting ZIKV replication and growth (Cui et al. 2020). In a virtual screening study targeting SARS‐CoV‐2 proteins, among 70 tea bioactive compounds, TFDG and TF2A exhibited the highest binding affinities towards three targets of SARS‐CoV‐2: RNA‐dependent RNA polymerase (RdRp), 3‐chymotrypsin‐like protease (3CLpro/Mpro), and papain‐like protease (PLpro), surpassing even Remdesivir and Favipiravir (Lung et al. 2020; Naidu et al. 2021). These compounds demonstrated a significant number of hydrogen bonds and other interactions both within and around the active sites of the three targets, which may affect the functional stability of viral proteins or block substrate binding sites, potentially impacting viral replication (Chang et al. 2022).

TFs play a crucial role not only in directly targeting various stages of viral infection but also in enhancing the body's antiviral capacity by modulating the host's immune response (Maiti et al. 2022; Chauhan et al. 2022). In vivo experiments have demonstrated that black tea extract (BTE) not only blocks the binding of viruses to host cells but also mitigates severe inflammation induced by SARS‐CoV‐2 by reducing matrix metalloproteinase (MMP), tumor necrosis factor‐alpha (TNF‐α), and free radicals. This indicates a significant protective effect on lung DNA and the extracellular matrix (Jokhadze et al. 2024). Furthermore, TF2B can notably alleviate pneumonia damage caused by influenza viruses (H1N1, H3N2, H5N1) by inhibiting the TLR4/MAPK/p38 pathway, decreasing the expression of inflammatory factors such as IL‐6 and TNF‐α, and increasing the survival rate of infected mice to 55.56% (Sima et al. 2023).

### 2.3 Cancer Prevention and Therapy

Cancer is identified as the second leading cause of global deaths, characterized by complex pathogenic mechanisms that involve the dysregulation of essential molecular regulators, which include enzymatic systems, growth modulators, transcriptional controls, and anomalies in signaling pathways (Cortés et al. 2020). Although they are effective for treatment, traditional oncological approaches such as chemotherapeutic drugs and ionizing radiation therapies often lead to significant iatrogenic complications and the development of drug‐resistant phenotypes (Lodi et al. 2025). While current epidemiological data do not provide conclusive evidence of a direct relationship between tea consumption and overall cancer risk (Hu, Yang, and He 2024; Hu, Li, et al. 2024), new clinical studies suggest that the regular consumption of black tea correlates with lower incidence rates of certain cancers, such as oral carcinoma (Xu et al. 2025) breast cancer (Zeinomar et al. 2023), ovarian cancer (Gao et al. 2019), and prostate cancer (Thomas et al. 2021). Recent progress in phytochemical research has recognized TFs, which are naturally occurring polyphenolic compounds from black tea, as potential therapeutic agents for tackling these clinical issues, due to their multitarget mechanisms of action and acceptable toxicity profiles (Lin, Chu, et al. 2024; See Table 2).

**TABLE 2:** Antitumor and cancer chemopreventive effect of TFs.

	Cancer	Conclusion	References
TFDG	Ovarian cancer	TFDG inhibits the proliferation of A2780/CP70 and OVCAR3 tumor spheres by suppressing cell viability and colony formation capacity TFDG targets ovarian cancer stem cells (CSCs) through Wnt/β‐catenin signaling pathway	Pan et al. (2018)
TFDG	Prostate cancer	TFDG reduces the proliferation of prostate cancer cells by influencing the PKCδ/aSMase signaling pathway, which is linked to the expression of the 67 kDa laminin receptor (67LR) TFDG suppresses tumor growth while enhancing the phosphorylation of PKCδ and the expression of aSMase in tumor xenograft models	Sun et al. (2022)
Black tea extract	HCT‐116 Colon cancer	Theaflavin suppress cell proliferation and tumor progression of HCT‐116 colon cancer cells and EAC‐induced solid tumors Theaflavin block activities of DNMT1 and DNMT3a in vitro and in vivo	Bhattacharya et al. (2020)
TFs	A375 Human melanoma cells	TF showed anti‐proliferative and pro‐apoptotic effects on A375 human melanoma cells in concentration‐dependent manner via P53 and JNK pathways TF markedly stimulated proteins associated with the P53 pathway (ATM, CHK1/2, P53, and CASP8/3) and those linked to the JNK pathway (ASK1, JNK, and C‐JUN) via phosphorylation and cleavage, leading to the activation of pro‐apoptotic factors (PARP, BAX, BIM, PUMA, and P53)	Zhang et al. (2020)
TFs	B16F10 melanoma cells	TFs exhibited antiproliferative, pro‐apoptotic, antimigrative, and tumor‐inhibitory effects on B16F10 melanoma cells in a dose‐dependent manner TFs upregulate the mRNA expressions of pro‐apoptotic genes and protein expressions of apoptosis‐related p53 and JNK signaling molecules and downregulate the protein expressions of proliferation‐related MEK/ERK and PI3K/AKT signaling molecules as well as the expressions of MMP2 and MMP9	Zhang et al. (2021)
TFDG	OVCAR‐3 human ovarian carcinoma	TFDG improves the phosphorylation of Chk2 to influence the balance of pro‐apoptotic and anti‐apoptotic Bcl‐2 family proteins, thereby triggering intrinsic apoptosis in a p53‐dependent manner TFDG elevates the expression of death receptors to stimulate extrinsic apoptosis in OVCAR‐3 human ovarian carcinoma cells The expression of p27 was increased by TF3, leading to G0/G1 cell cycle arrest in OVCAR‐3 cells	Gao et al. (2019)

TFs exhibited their antitumor properties through complex mechanisms including antioxidant, proapoptotic, antiproliferative, antiangiogenic, immunomodulatory, and antimetastatic activities (O'Neill et al. 2021). As robust antioxidants that feature several phenolic hydroxyl groups, TFs could efficiently neutralize free radicals like superoxide anions, trigger the intracellular antioxidant defense system, sustain cellular redox equilibrium, and lower the risk of cancer ^[19]^. In addition to their antioxidant capabilities, TFs also induce apoptosis in tumor cells. This occurs through the activation of apoptotic signaling pathways and alteration in the expression of proteins associated with apoptosis, a process that favors programmed cell death (He et al. 2022). Furthermore, TFs are instrumental in disrupting the tumor cell cycle and inhibiting critical signaling pathways that are linked to proliferation. This results in a suppression of tumor cell division and growth (Pal et al. 2017). TFs also play a significant role in targeting angiogenesis, a vital process for tumor growth and metastasis. They inhibited both the expression and the activity of vascular endothelial growth factor (VEGF) as well as matrix metalloproteinases (MMPs), which were essential for the formation of new blood vessels that supply tumors (Zhang, Dai, et al. 2018; Zhang, Suen, et al. 2018). Moreover, TFs are known to enhance immune function by stimulating various immune cells, including macrophages, natural killer (NK) cells, and cytotoxic T lymphocytes (CTLs). They also regulate the secretion of key cytokines such as interleukin‐2 (IL‐2) and interferon‐γ (IFN‐γ), promoting the establishment of an antitumor immune microenvironment (Roy et al. 2021) Additionally, TFs have been shown to inhibit the activity of adhesion molecules and MMPs located on the surfaces of tumor cells. This disruption, combined with the inhibition of intracellular signaling pathways mediated by Rho family GTPases, leads to comprehensive suppression of tumor cell invasion and metastasis (Sun et al. 2022).

### 2.4 Antibacterial Activity

Research has indicated that TFs exert a distinct inhibitory influence on various types of Gram‐negative and Gram‐positive bacteria, as well as fungi associated with human diseases (Yussof et al. 2022). Their effects encompass direct antibacterial properties, enhancement of antibiotic efficacy, and the suppression of bacterial virulence (see Table 3). The understanding of how TFs work to inhibit harmful microorganisms remains incomplete; however, researchers suggest that the hydroxyl group on the benzophenone ring plays a crucial role in influencing their antibacterial effectiveness. In a manner similar to catechins, TFs' antibacterial properties might be linked to their interactions with bacterial membranes, leading to irreversible damage and potentially altering the characteristics of the cell membranes in pathogenic bacteria (Luo, Nie, et al. 2024).

**TABLE 3:** Antibacterial effects of theaflavins and their derivatives.

TFs	Bacteria	Conclusions	References
TFDG	Nine bacteria and the sporicidal	TFDG prevents the growth of nine types of bacteria, including Gram‐positive, Gram‐negative, and acid‐fast strains, achieving growth inhibition of up to 99.97% TFDG treatment reduced the level of gpr gene expression in spores of both *Bacillus cereus* and *B. subtilis*	Yussof et al. (2022)
TFDG	*Listeria monocytogenes*	TFDG showed excellent antibacterial activity against *L. monocytogenes* with the minimum inhibitory concentration of 62.5 mg L^−1^	Lin, Chu, et al. (2024), Lin, Shen, et al. (2024)
TFs	*Bacillus coagulans*	TFs exhibit high bactericidal activity at 50 μmol L^−1^, whereas EGCG does not Treatment with TFs decrease cell membrane fluidity of *B. coagulans*	Sato et al. (2022)
TFDG	*Clostridium perfringens*	TFDG had twice as strong antibacterial activity as EGCG against *C. perfringens*	Mohamed et al. (2022)
TFDG	*Bacillus coagulans*	The bactericidal activity of TFDG against *B. coagulans* was observed at concentrations exceeding 62.5 mg L^−1^, whereas EGCg did not exhibit such effects at same concentration After treatment with TFDG at a concentration of 62.5 mg L^−1^, the glucose transporter activity in the cells diminished by 40%	Sato et al. (2020)
TFDG	Gram‐positive and Gram‐negative pathogens	TFDG inhibits the hydrolysis of Metallo‐β‐lactamases (MBLs) The binding of TFDG to Gln242 and Ser369 is observed to create a synergistic effect with β‐lactam antibiotics against methicillin‐resistant *Staphylococcus aureus* BAA1717, thereby enhancing the antibacterial activity of these antibiotics against pathogens both in vitro and in vivo	Teng et al. (2019)

### 2.5 Other Activity

Epidemiological studies have demonstrated significant cardiovascular benefits associated with black tea consumption (Pan et al. 2022). Robust computational modeling approaches, including molecular docking, free energy calculations, and molecular dynamics simulations, have identified TFs as putative agonists of key molecular targets, potentially elucidating their observed hypocholesterolemic effects in tea (Adigun et al. 2023). In vitro experiments have shown that TF‐1 significantly inhibits platelet aggregation in a dose‐dependent manner while attenuating key markers of platelet activation, including P‐selectin surface expression, fibrinogen receptor binding, and thromboxane A2 (TxA2) biosynthesis (Zhang et al. 2022).

Furthermore, black tea has long been utilized in traditional folk medicine as a remedy for managing hyperglycemia (Luo et al. 2023). In vivo experiments have clearly demonstrated that TFs could effectively mitigate hyperglycemia in diabetic mice by inhibiting α‐amylase, with TFDG emerging as the most potent bioactive component (Li, Li, et al. 2024). Moreover, TFs have exhibited the ability to ameliorate glycolipid metabolism disorders in diabetic patients, contributing not only to the reduction of blood sugar and lipid levels but also to the alleviation of various types of damage, such as glucotoxicity, lipotoxicity, and other secondary adverse effects associated with diabetes (Xu, Chen, and Gong 2024; Xu, Zeng, et al. 2024).

## 3 Formation Mechanism of TFs

In 1959, Roberts and Myers (1959) successfully isolated TFs from black tea and elucidated their structures. They proposed that TFs arise from the coupled oxidation reactions of epicatechin gallate (EGC) and epigallocatechin gallate (EGCG). Since that time, numerous researchers have conducted extensive studies on the molecular structure of TFs and the variations among different catechins during enzymatic reactions. Building upon these foundational findings, researchers have proposed potential mechanisms and pathways for the formation of theaflavins (Kusano et al. 2015; Takino and Imagawa 1963; Bailey et al. 1993). A widely accepted pathway for TF formation is illustrated in Figure 2 and described as follows.

**FIGURE 3:**
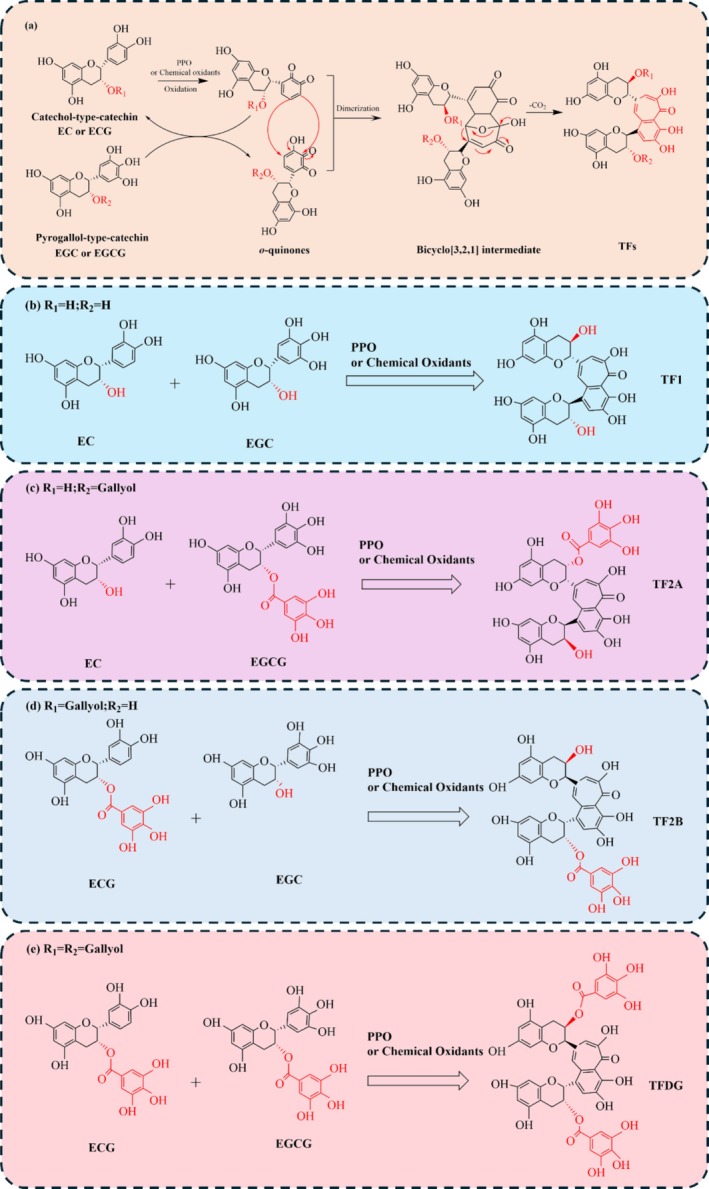
(a) The formation mechanism of PPO‐catalyzed theaflavin from catechins; pathways for the synthesis of four main theaflavins: (b) TF1; (c) TF2A; (d) TF2B; (e) TFDG.

Catechins, as precursors to theaflavin synthesis, can be classified into two categories based on the structure of the B ring: catechol‐type and pyrogallol‐type catechins (Jian et al. 2024). The catechol‐type catechins primarily include epicatechin (EC) and epigallocatechin (EGC), whereas the pyrogallol‐type catechins mainly consist of catechin gallate (ECG) and epigallocatechin gallate (EGCG). The key distinction between these two types lies in the presence of a galloyl group on the B ring of the catechin. Catechol‐type catechins, which lack a galloyl group on the B ring, exhibit a lower redox potential, making them more susceptible to oxidation to EC‐quinone under the catalytic influence of polyphenol oxidase (PPO) during enzymatic reactions (Jin et al. 2021). EC‐quinone subsequently serves dual functions: as an oxidant and as an electrophilic enone (Long et al. 2023). As an oxidant, EC‐quinone reacts with pyrogallol‐type catechins, oxidizing their B ring to form O‐quinone while being reduced back to catechol‐type catechins (Tanaka and Matsuo 2020). More importantly, EC‐quinone can act as an electrophilic enone and undergo a cycloaddition reaction with the electron‐rich B ring of pyrogallol‐type catechins (Matsuo and Tanaka 2017). To maintain aromaticity, a bicyclo[3.2.1]octane‐type intermediate (BOI) is formed through rearrangement. Subsequent rapid oxidation and decarboxylation of BOI generate theaflavin (TF) in the presence of water (Matsuo et al. 2009). According to the aforementioned mechanism, the four main theaflavins are TF1, TF2A, TF2B, and TFDG. They are generated from the coupling reactions between EC and EGC, EC, and EGCG, EGC and EGC, and EGC and EGCG (Lian et al. 2024), respectively, as shown in Figure 3.

The results of model fermentation and the structural study of various new tea polyphenol oxidation products provide compelling evidence for the formation mechanism of theaflavins (Chen, Wang, et al. 2023; Chen, Wei, et al. 2023; Wang, Ren, Chen, Lu, et al. 2023). However, certain issues with the aforementioned models necessitate further investigation. For example, due to the complexity of the synthesis reactions and the involvement of various unstable intermediates, the detailed generation mechanism of BOI, a crucial intermediate in the formation of TF, remains unclear. Furthermore, the structures of additional condensation products of TFs, specifically thearubigins (TRs) and theabrownins (TBs), have yet to be clearly defined, and the mechanism by which TFs are further transformed into TRs and TBs remains uncertain. Addressing these issues through further research is essential to provide theoretical and empirical support for the efficient preparation of theaflavins.

## 4 Research Progress of TF Synthesis Methods

As the efficacy of TFs beneficial to human health continues to be substantiated, the demand for large‐scale synthesis of TFs is becoming increasingly urgent. Currently, the primary source of TFs is extraction from black tea (Tang et al. 2023). However, TFs' content in black tea is notably low, and the extraction and purification processes are challenging and costly, resulting in an insufficient supply of high‐purity TFs to meet product development needs (Dong et al. 2025). With ongoing advancements in TFs research, in vitro simulated oxidation methods are emerging as the most promising preparation techniques for TFs. These methods can be categorized into enzymatic and chemical oxidation methods based on the different catalysts employed (Zhao et al. 2025).

### 4.1 Enzymatic Oxidation

The distribution of polyphenol oxidase (PPO) and catechin in tea occurs in distinct cellular compartments; PPOs are primarily located in the cell wall, while catechin is predominantly found in the cell fluid (Guo et al. 2025). Consequently, the production of TFs during black tea processing takes place only after the disruption of the cell structure, which facilitates the interaction among PPO, catechin, and oxygen. This compartmentalization is the key factor contributing to the low levels of TFs in black tea. The enzymatic oxidation method involves the catalysis of catechin oxidation by polyphenol oxidase (PPO) and other oxidoreductases in an oxygen‐rich environment outside the tea leaves. This approach effectively addresses the challenge of ensuring contact among the reaction precursor catechins, PPO, and oxygen during black tea fermentation. By optimizing reaction conditions, this method can significantly enhance both the reaction rate and the purity of TFs, ultimately reducing production costs (Tanaka et al. 2002). Currently, the primary enzymatic oxidation methods include homogenate suspension synthesis (Jiang et al. 2018), biphasic system enzymatic synthesis (Narai‐Kanayama et al. 2018), and immobilized enzyme synthesis (Tu et al. 2005).

#### 4.1.1 Enzyme Source Screening

According to the enzymatic oxidation mechanism of TFs, two key oxidases drive the catalytic conversion of catechins to o‐quinones. PPO mediated the oxygen‐dependent oxidation of catechins, while peroxidase (POD) facilitated hydrogen peroxide‐driven oxidation and the subsequent polymerization of TFs into thearubigins (TRs) and theabrownins (TBs) (Long et al. 2023). Notably, total TF content exhibited a direct positive correlation with enzyme activity levels. Increased PPO activity specifically indicates an enhanced catalytic capacity for catechin oxidation and TF biosynthesis (Chiang et al. 2022). Therefore, the selection of high‐activity enzyme sources is a critical determinant in optimizing the enzymatic synthesis of TFs.

Although tea contained a significant amount of endogenous PPO, the composition of isoenzymes and substrate catechins associated with these PPOs varies considerably due to endogenous factors such as variety and location, as well as exogenous factors including environmental conditions and climate. Consequently, tea fermentation is a complex catalytic process. Analyzing and purifying the crude PPO enzyme in tea not only aided in elucidating the mechanism of catechin enzyme‐catalyzed oxidation but also enabled the acquisition of highly efficient PPO for the targeted conversion of TFs (Liu, Chen, Liu, Li, et al. 2022). A seminal study by Teng et al. (2017) demonstrated this functional divergence through the multistep chromatographic purification of two PPO isoenzymes (PPO1 and PPO2) from *Camellia sinensis* var. Zhenghedabai leaves, achieving a 48.94‐fold purification. Biochemical characterization revealed that PPO1 exclusively catalyzes the synthesis of theaflavin (TF1), while PPO2 exhibits a broader catalytic capacity, generating four distinct TF monomers, with TFDG demonstrating the highest conversion efficiency. Traditional purification strategies utilize sequential techniques such as fractional salting‐out (e.g., ammonium sulfate), dialysis, and coupled chromatography (Teng et al. 2014). Among these methods, ion exchange, gel filtration, affinity, and hydrophobic chromatography facilitate selective separation based on the molecular characteristics of PPO (size, charge, ligand affinity; Tang et al. 2023). Notable examples include Öztürk et al.'s (2020) implementation of single‐step affinity chromatography using Sepharose 4B‐L‐tyrosine‐p‐aminobenzoic acid, which achieved a 19.77‐fold PPO purification with a specific activity of 406,091 EU/mg and a recovery of 2.67%. Recent advancements in three‐phase partitioning (TPP), which integrates extraction, separation, and purification, attained a 15.76‐fold PPO purification (specific activity: 210.46 U/mg, recovery: 8.04%) using (NH_4_)_2_SO_4_/t‐butanol (Liu, Chen, Liu, et al. 2022). Furthermore, substituting t‐butanol with the deep eutectic solvent DES‐7 (thymol/dodecanoic acid) enhanced recovery to 78.44% with an 8.26‐fold purification (Xu, Chen, and Gong 2024; Xu, Zeng, et al. 2024).

Current research indicated that the enzymatic characteristics and catalytic efficiency of tea polyphenol oxidase (PPO) were significantly influenced by various factors, including plant cultivar, seasonal variations, and environmental growth conditions (Li et al. 2021). The high costs and limited availability of tea endogenous enzymes posed challenges in meeting the demands of industrial production, highlighting the necessity for alternative enzyme sources. In addition to tea, PPO is widely distributed among various plants and microorganisms (McLarin and Leung 2020). Notably, plant‐derived PPO exhibits greater sequence homology with endogenous tea PPO compared with its microbial counterparts, making it particularly suitable for catalyzing the biosynthesis of TFs (Zou et al. 2025). In a comprehensive screening study, Tanaka et al. (2002) evaluated 62 plant tissue homogenates and identified 46 species capable of catalyzing the synthesis of TF1. Remarkably, loquat ( *Eriobotrya japonica* ), Japanese pear ( *Pyrus pyrifolia* ), and blueberry (Vaccinium spp.) demonstrated even higher enzymatic activity than fresh tea leaves in this catalytic process. Consequently, there was growing scientific interest in identifying and optimizing PPO enzymes from nontea sources that can maintain consistent catalytic performance under controlled production conditions. Such efforts aimed to develop reliable biocatalytic systems capable of supporting the industrial‐scale production of TFs required for nutraceutical and pharmaceutical applications. In recent years, an increasing number of studies have demonstrated the application of exogenous PPO in the synthesis of TFs, thereby enriching the sources of PPO enzymes (see Table 4).

**TABLE 4:** Comparison of enzymatic synthesis of theaflavins by PPO from different sources.

PPO sources	Temperature	PH	Theaflavin content/conversion rate	References
Potato	30	6.0	22.86%	Li and Li (2021)
Fengshui pear Laccase Hongfushi apple	37	—	0.228 mg/mL 0.011 mg/mL 0.008 mg/mL	Xue et al. (2019)
Potato	20	5.5	0.657 mg/mL	Tang et al. (2023)
Bergamot yam	35	5.5	30.66% (TF1)	Li et al. (2023)
Sweet potato wastewater	25	6.0	214.3 μg/mL	Li et al. (2018)
Pear	40	5.0	24.47 mg/g	Li, Bai, et al. (2022), Li, Yuan, et al. (2022)

#### 4.1.2 Optimization of Reaction System

According to the synthesis mechanism of TFs, the enzymatic synthesis reaction of TFs is a complex heterogeneous process. External reaction conditions, such as pH, reaction temperature, reaction time, and catechin concentration, can significantly affect the yield of TFs, as these factors influence enzyme activity, dissolved oxygen levels, and the redox potential of the substrate catechin.

The pH value of the reaction system influences the synthesis of theaflavins by affecting enzyme activity as well as the stability of substrates and products. Although the optimal pH range for polyphenol oxidase (PPO) enzymes differs from various sources, all fall within the acidic range of pH < 6. Both excessively high and low pH values can lead to a reduction in enzyme activity (Wang, Ho, and Li 2022). Lowering the pH of the reaction system can also modify the redox potential of catechins, accelerating their oxidation rate and consequently increasing the total formation of theaflavins (An et al. 2023). For instance, Teng et al. (2021) utilized tea leaf PPO isoenzymes as the enzyme source and discovered that at a pH of 5.0, the yield of TFs reached a maximum of 2.84 μg/mL. However, when the pH exceeded 6.5, the yield of TFs significantly decreased. In contrast, when employing potato PPO ( *Solanum tuberosum* L.) as the enzyme source, the optimal pH was identified as 5.0, with a notable decrease in TF yield when the pH exceeded 7.0 (Kong, Xu, et al. 2023).

The reaction temperature is also a critical factor influencing the activity of PPO and the subsequent formation of TFs (Adeseko et al. 2021). Most plant‐derived PPOs, including those found in tea leaves, exhibit peak activity at temperatures ranging from 30°C to 35°C, which facilitates the efficient oxidation of catechins (e.g., EGCG, ECG) into TFs during the enzymatic reaction. For instance, the optimal activity of internal PPO from tea is observed at 30°C–40°C, while microbial‐derived PPOs, such as those from Aspergillus spp., show optimal activity at 35°C. In contrast, the optimal temperature for potato and pear PPO is 20°C (Hua et al. 2018). When the reaction temperature falls below 20°C, the reduced enzyme activity slows catechin oxidation and delays the formation of theaflavins, although this may yield higher TFs (Ding et al. 2019). Conversely, when the reaction temperature exceeds 40°C, rapid denaturation of PPO occurs, leading to a diminished yield of theaflavins. For example, Liubao tea PPO loses 77% of its activity at 70°C within 5 min (Long et al. 2022). Therefore, the optimal reaction temperature for the enzymatic synthesis of TFs is generally below 30°C. For instance, when using potato polyphenol oxidase (PPO) from *Solanum tuberosum* L. as the enzyme source, lower temperatures are more favorable for the formation of tea yellow pigments, with the best results achieved at 20°C.

In the initial stage of synthesizing TFs, the yield increases rapidly with prolonged reaction time. However, as the reaction time extends further, the yield stabilizes or gradually decreases. The optimal reaction time varies between 30 and 120 min, depending on the reaction temperature and pH, and tends to shorten as the temperature rises. For example, when using potato polyphenol oxidase (PPO) derived from *Solanum tuberosum* L. as the enzyme source, the inflection point occurs at 60 min at 20°C, while it occurs at 30 min at 40°C.

The ratio and addition method of catechin monomers also significantly influences the formation of theaflavins. For example, when the ratio of EGC to EGCG is 1:2, the yields of TF2B and TFDG are relatively high, which significantly enhances the total content of TFs. Incremental addition of catechin substrates promotes a more uniform, thorough, and efficient oxidation of EGC and EGCG, thereby maintaining a high conversion rate of TFs (Hua et al. 2021).

Based on the analysis presented, the optimal reaction conditions for the majority of enzymatic syntheses of TFs are identified as follows: a fermentation temperature ranging from 20°C to 30°C, a pH level between 5.0 and 6.0, and a fermentation duration of 30–60 min.

#### 4.1.3 Enzyme Engineering Optimization

Enzymes serve as the primary catalysts for the synthesis of TFs through enzymatic oxidation. However, the economic cost associated with this process remains high. This is largely due to the poor stability of natural free enzymes, which are prone to loss, nonrecovery, and the elevated costs of extraction and separation. In recent years, enzyme engineering techniques, such as the use of immobilized enzymes and recombinant enzymes, have emerged as promising strategies for producing stable, efficient, and cost‐effective polyphenol oxidases (Wu et al. 2021).

Enzyme immobilization technology involves the process of immobilizing free enzymes within solid materials or confining their catalytic action to specific areas through physical or chemical means. The primary methods for enzyme immobilization include adsorption, embedding, carrier coupling, and cross‐linking methods. Immobilized enzymes not only enhance enzyme stability but also offer advantages in recovery and reusability, significantly improving the economic viability of enzymatic oxidation synthesis of TFs (Fei et al. 2022). Liu et al. (2020) compared the effects of the sodium alginate embedding method and the glutaraldehyde cross‐linking method on the enzymatic properties of polyphenols. The results indicated that the immobilized enzyme exhibited superior pH stability and thermal stability compared with the free enzyme. Although the glutaraldehyde cross‐linking method resulted in a greater loss of enzyme activity (50%), its enzyme activity collection rate (24.0%) was notably higher than that of the sodium alginate embedding method. Sharma et al. (2009) reported that immobilized enzymes were produced using the coupling method with cellulose as a carrier, achieving an enzyme activity retention of 83.58% and allowing for 14 batches per turnover without loss of activity. When employed for the synthesis of TFs, the total conversion efficiency reached 85%, which is 14 times greater than the maximum yield from normal black tea.

Native PPO typically comprises a mixture of various isoenzymes, and increasing its yield presents challenges that impede further development of applications (Ye et al. 2021). However, advances in DNA recombinant technology, large‐scale biological fermentation, and techniques for the isolation, purification, and extracellular export of recombinant proteins have revealed significant commercial potential for recombinant PPO (Li, Shen, et al. 2024). Currently, *Escherichia coli* , yeast, and filamentous fungi are the most utilized hosts for investigating the heterologous expression of recombinant PPO. Zeng et al. (2019) successfully subcloned PPOs from nine plant species into the pET32a(+) vector and expressed them in *E. coli* BL21(DE3). LC–MS‐based enzyme assays demonstrated that eight recombinant PPOs exhibited catalytic activity in converting tea polyphenols into TFs (TFs), with notably higher efficiency observed for Md2 from *Malus domestica* (apple), Pp4 from Pyrus pashia (pear), and Ej2 from *Eriobotrya japonica* (loquat). Following this, Cai et al. (2023) expanded this research by cloning two PPO isozymes (HjyPPO1 and HjyPPO3) from *Camellia sinensis* cv. Huangjinya into pET28a(+) and expressing them in the same bacterial system. Both isozymes catalyzed catechin oxidation to produce four TFs, with HjyPPO3 demonstrating superior catalytic efficiency compared to HjyPPO1. To optimize the expression of CsPPO in *E. coli* , Singh et al. (2017) cloned a codon‐optimized version of CsPPO into the pET‐47b(+) vector. The initial expression trials were conducted in *E. coli* BL21(DE3), followed by *E. coli* Rosetta2, using 1.0 mM isopropyl β‐D‐1‐thiogalactopyranoside (IPTG) for induction. Extensive optimization of buffers and refolding methods was performed, including dialysis, on‐column refolding, and the use of a rapid dilution buffer containing 0.5 M L‐arginine. The refolded CsPPO exhibited optimal activity at pH 5.0, with *K*_m_ values of 3.10, 0.479, and 0.314 mM, and corresponding *V*_max_ values of 163.9, 82.64, and 142.8 U/mg of protein for catechol, catechin, and epicatechin, respectively.

### 4.2 Nonenzymatic Synthesis

Since the content of TFs in black tea is very limited, it is very difficult to obtain the TFs required for research. In 1963, Takino and Imagawa (1963) found that the reaction of EC and GC with potassium ferrocyanide and sodium bicarbonate can obtain the same tea pigments as TFs in tea. Chemical oxidation mainly uses inorganic oxidants to oxidize catechins to obtain TFs (Qu and Ma 2006). Compared with enzymatic oxidation, chemical oxidation offers a simpler reaction process and greater ease in controlling reaction conditions. More importantly, it addresses challenges associated with enzyme extraction and purification, the instability of enzyme properties, and factors such as oxygen restriction. Consequently, chemical oxidation presents a viable alternative method for the early in vitro preparation of TFs (Liu et al. 2024). The chemical oxidation method can be categorized into two types based on the nature of the oxidants: acidic oxidation and alkaline oxidation (Chang et al. 2024). The acidic oxidation method primarily employs acidic oxidants, such as ferric nitrate and ferric chloride, while the alkaline oxidation method utilizes alkaline oxidants like potassium ferricyanide and sodium bicarbonate. A notable advantage of the alkaline oxidation method is its significantly higher production of TFs compared with the acidic method (Luo et al. 2017). Under identical reaction conditions, the yield of TFs in the alkaline method could exceed that of the acidic method by more than 2‐fold (Zhang et al. 2011a). However, the reactions involved in the alkaline oxidation method are more complex and tend to generate a greater number of by‐products, which complicates subsequent purification processes (Zhang et al. 2011b). The use of strong oxidants, such as high‐valent iron salts, in chemical oxidation methods raises environmental concerns and complicates the purification of TFs. However, these chemical oxidation methods offer a more direct and highly reproducible approach for the synthesis studies of TFs (Ding et al. 2020).

Biomimetic synthesis employed chemical methods to replicate the processes of organisms or enzymes for organic synthesis, thereby offering innovative approaches for the preparation of naturally active ingredients. This method is characterized by its high efficiency and selectivity. According to the formation mechanism of TFs, the condensation of catechins with o‐quinone to produce bicyclo[3.2.1] octane intermediates was the key step in forming the benzotropolone characteristic structure of TFs (Wang, Guo, et al. 2025; Wang, Zhao, et al. 2025). Numerous studies have been conducted on the biomimetic synthesis of bicyclo[3.2.1] octane intermediates. Yanase et al. (2005) employed 4‐methylionone and 5‐methylthioanisole as simplified analogues of catechins. In the aprotic solvent dichloromethane, these compounds were oxidized using the Fetizon reagent (Ag_2_CO_3_ and diatomaceous earth), resulting in the formation of the bicyclic[3.2.1] intermediate with an optimal yield of 94%. Kawabe et al. (2013) utilized lead tetraacetate (Pb(OAc)4) as an oxidizing agent and acetonitrile as a solvent to achieve the biomimetic synthesis of TFs from catechin at 0°C. The synthetic reaction employed 2‐nitrobenzenesulfonyl (Ns) to mitigate side reactions of electron‐rich aromatic rings, thereby facilitating the one‐step synthesis of benzophenones. Similarly, Matsuo et al. (2017) employed DPPH as an oxidizing agent to develop a novel method for the nonenzymatic biomimetic synthesis of TFs without the use of a protective group, achieving a maximum efficiency of 47%.

## 5 Future Challenges and Perspectives of TFs Synthesis

### 5.1 Future Challenges

**FIGURE 4:**
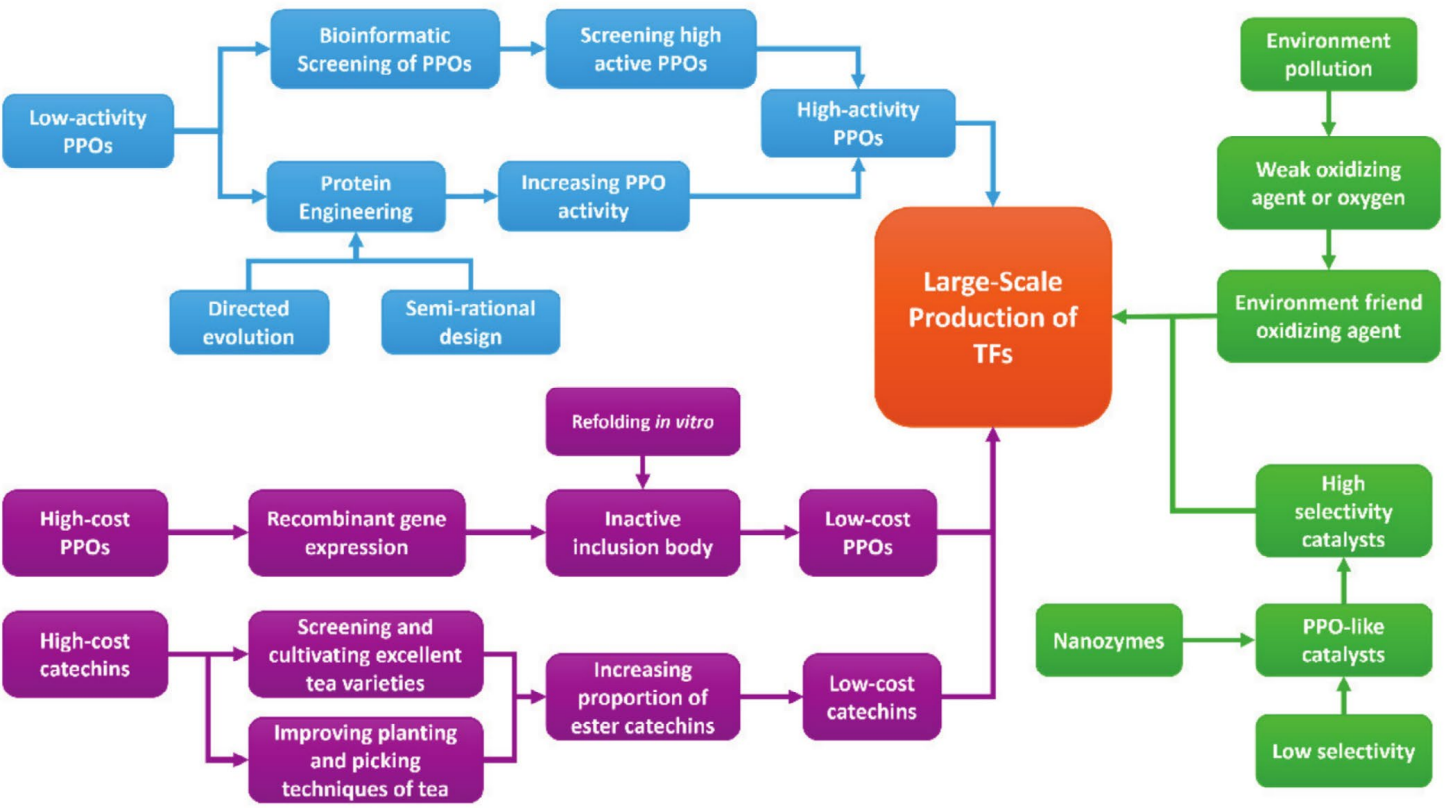
Proposed research strategies to large‐scale production of TFs.

Although significant progress has been made in the synthesis of TFs in recent years, numerous challenges remain that must be urgently addressed for industrial applications (see Figure 4). These challenges primarily focus on the following aspects:

#### 5.1.1 Due to Low Bioavailability and Stability, The Physiological Activities Obtained From Cell and Animal Experiments Cannot Be Directly Extrapolated to Humans

Despite the significant attention and substantial progress in research on the physiological activities of theaflavins in recent years, there are currently very few food or pharmaceutical products that utilize theaflavins. This scarcity is primarily due to low bioavailability and stability.

Research indicates that the bioavailability of TFs in the human body is relatively low. Two hours after the intake of 700 mg of theaflavins, the maximum concentration of 1 μg·mL^−1^ can be detected in plasma, while in urine, a concentration of 4.21 μg·mL^−1^ of theaflavins can be observed (Mulder et al. 2001). This low bioavailability may be attributed to the presence of numerous hydrophilic hydroxyl groups in TFs, which hinder their passage through lipid bilayers. Additionally, the large molecular weight of TFs and the lack of active transporters for TFs in cells result in an absorption mechanism that relies solely on passive diffusion. Consequently, the intestinal stability of theaflavins is poor, leading to a low absorption rate in the small intestine, with only a minimal percentage of the ingested amount being utilized by the human body, estimated at only 0.001% of the intake (El‐Saadony et al. 2024; Pereira‐Caro et al. 2017). Currently, most research on the physiological activity of TFs is confined to cellular or animal experiments, with various mechanisms proposed to explain their physiological activity based on cellular targets and physical structures. However, since theaflavins are not systemically bioavailable, the mechanisms proposed from cellular or animal studies may be irrelevant in vivo due to the differences in the concentrations of TFs, cell types, and cellular environments used (Yang et al. 2025).

The structure of tea yellow monomers (TFs), characterized by a benzophenone framework, consists of three main components: a fused structure of a benzene ring and a seven‐membered diene cycloketone, and two fused structures of benzene rings with tetrahydropyran (Li et al. 2013). The structure of theaflavins indicates their susceptibility to external environmental conditions such as pH, high temperatures, and light, leading to oxidation, decomposition, and tautomeric interconversion (Yu et al. 2022). Research has confirmed that theaflavins exhibit poor stability in temperature, light, pH, and oxygen when in aqueous solution or under very humid storage conditions of black tea extracts. For instance, when heated at 80°C for 30 min, the decomposition rates of the four main theaflavin monomers approached 60%. Under alkaline conditions with pH > 8, the decomposition rate of theaflavin monomers also exceeds 40% (Su et al. 2003). During the preparation, extraction, storage, and application of TFs, the influence of external factors such as temperature, acidity, ions, and air is unavoidable. Therefore, it is essential to conduct comprehensive and systematic studies on the stability of TFs, particularly concerning degradation products under various conditions. However, research in this area remains limited.

Furthermore, since theaflavins are naturally derived compounds and black tea has been consumed as a daily beverage for a long time, they are generally considered to be nontoxic or of low toxicity. Acute toxicity studies on black tea and theaflavin extracts (with a purity of less than 60%) have also confirmed that the acute oral median lethal dose (LD_50_) for both female and male rats is greater than 2 g/(kg BW), which is more than 100 times the adult daily intake. Therefore, according to the WHO classification standards for compound toxicity, theaflavin extracts are categorized as low toxicity (Zhang et al. 2014).

Further research has demonstrated that the results of the Ames test, mouse bone marrow micronucleus test, and sperm deformity test were all negative. A 30‐day feeding trial also confirmed that there were no abnormalities in rat body weight, food intake, food utilization rate, hematological indices, terminal blood biochemical indices (except for blood glucose), and organ‐to‐body weight ratios (Kong, Diao, et al. 2023). These findings collectively support the safety of theaflavins and their extracts for use in food and personal care products. However, the safety of polyphenolic substances such as theaflavins may vary based on purity composition, exposure routes, and methods of intake. For example, injecting EGCG can result in severe liver damage in experimental animals, while feeding and oral administration have shown minimal impact (Zhao et al. 2022). Therefore, the current findings regarding the safety of theaflavins cannot be directly extrapolated to their medicinal applications.

However, the limited industrial production of high‐purity TFs results in most theaflavin products being mixtures of various theaflavin monomers, characterized by high costs and low purity. This situation significantly hinders the research and application of TFs. Consequently, research focused on the preparation and purification processes of theaflavins that are low‐cost, high‐purity, and environmentally friendly represents a critical challenge in addressing the current limitations in the field.

#### 5.1.2 The Most Significant Challenge for Enzymatic Oxidation Remains the Discovery of Enzyme Sources That Are Cost‐Effective, Stable, and Efficient

Despite the widespread presence of PPO in nature, plant‐sourced PPO has fundamental limitations, including challenges in preservation and unstable enzyme characteristics (Chen et al. 2025). The activity of PPO derived from microbes in catalyzing catechins is relatively low, which suggests that natural PPO is not an optimal enzyme source for large‐scale enzymatic oxidation aimed at synthesizing TFs. Recombinant PPO enzymes could represent a promising approach for the industrial production of PPO (Cronin and Yu 2023); however, the prokaryotic nature of *E. coli* prevents the occurrence of essential post‐translational modifications, such as the formation of disulfide bonds, phosphorylation, glycosylation, and fatty acid acylation in proteins. Consequently, PPO is frequently produced in *E. coli* as inactive, insoluble inclusion bodies, creating significant obstacles for generating soluble recombinant PPO with the desired biological functions at both laboratory and industrial scales (Gan et al. 2018).

#### 5.1.3 Reactions That Are Both Green and Highly Selective Represent the Primary Focus of Nonenzymatic Synthesis

Reactions that are both green and highly selective represent the primary focus of nonenzymatic synthesis. In contrast to enzyme‐catalyzed methods, nonenzymatic synthesis offers specific benefits regarding conversion rates, reproducibility, and cost‐effectiveness. Nonetheless, notable challenges remain concerning selectivity and environmental impact, which hinder its widespread industrial use. Currently, chemical oxidation predominantly employs strong oxidizers like ferric salts, leading to the generation of numerous by‐products and significant environmental issues. Biomimetic catalysis addresses the challenges related to reaction selectivity and demonstrates clear advantages in both conversion efficiency and product purity. However, it often involves multiple reaction steps, prolonged processes, or the use of particular hydroxyl‐protecting agents, which complicate its feasibility for large‐scale industrial applications.

#### 5.1.4 Appropriate Raw Materials for Tea Can Facilitate the Large‐Scale Synthesis of TFs

Appropriate raw materials for tea can facilitate the large‐scale synthesis of TFs. Currently, numerous studies focus on pure catechins as their subjects. Clearly, utilizing them directly for large‐scale production could significantly escalate costs. Research findings indicate that the composition of catechins is closely linked to that of TFs. Thus, not every variety of tea is appropriate for the synthesis of TFs. Therefore, identifying tea tree varieties that possess an ideal catechin composition will be a key challenge for future industrialization.

### 5.2 Perspectives

#### 5.2.1 Proposed Research Strategy for Enhancing Bioavailability and Stability

Although the in vitro physiological activity of TFs has been widely confirmed, the poor bioavailability and stability of theaflavins make it difficult to achieve effective therapeutic concentrations through oral intake (Yin et al. 2022). This limitation significantly restricts their application in fields such as food and medicine.

Encapsulation and drug delivery systems, such as emulsions, nanoparticles, microcapsules, and hydrogels, have been applied to enhance the bioavailability and stability of bioactive substances or improve their applicability in various industries, including food, chemical, casting, medical, and pharmaceutical (Huang et al. 2025). Research has confirmed that encapsulating theaflavins in Pickering emulsions significantly enhances their bioavailability and stability, increasing physiological bioavailability from 17.7% in the homogeneous aqueous solution to 57.8%. After storage for 21 days and heat treatment at 105°C for 4 h, the retention of theaflavins in the Pickering emulsion is 1.4‐fold higher than that in the homogeneous aqueous solution (Lu, Wu, et al. 2024). Encapsulation of theaflavins using chitosan (CS)‐based polysaccharide–peptide nanocomplexes (GCCNs) has also been shown to significantly improve the in vitro intestinal permeability of TFDG. The total amount of transported TFDG encapsulated in GCCNs increases by a factor ranging from 7.8 to 9.5‐fold compared to that of pure TFDG (Jiang et al. 2023).

Nanoconjugation refers to the process of attaching biomolecules, such as proteins, nucleic acids, or natural compounds, to the surfaces of nanomaterials (Malik et al. 2024). This technique has been established as an effective strategy for various biological applications and drug delivery. Due to their capability to enhance therapeutic safety and efficacy by targeting specific sites within the body, nanoconjugates have garnered increasing attention for their potential to improve stability, solubility, and controlled release conditions (Al‐Shimmary et al. 2025). The formation of the nanoconjugate AuNP@TfQ, which is synthesized by conjugation of theaflavins with gold nanoparticles (AuNPs), has been confirmed to significantly enhance its apoptotic ability compared to pure TFs, with the half‐maximal inhibitory concentration (IC_50_) for ovarian cancer cells decreasing from 56 to 11 μg/mL (Maity et al. 2019).

However, the results of these studies are still limited to the cellular level, and the effectiveness in the human body remains a challenge. For the oral administration route, encapsulated emulsions, nanoparticles, and other formulations must withstand the harsh pH and digestive enzyme environment in the gastrointestinal tract while maintaining the payload of polyphenols to reach the drug absorption site in the small intestine. Additionally, attention should be paid to the impact of nanoencapsulation on the metabolism of polyphenols and its effects on relevant metabolic enzymes to further elucidate the mechanisms behind the enhanced bioavailability following nanoencapsulation (Hu et al. 2017).

#### 5.2.2 Proposed Research Strategy for Enzyme Source Screening

In recent years, there has been a growing identification of natural polyphenol oxidases (PPOs) that can catalyze the formation of TFs. Nevertheless, these PPOs are frequently hard to fulfill the demands for industrial production, especially concerning their stability and yield (Zou et al. 2025). Hence, it is crucial to investigate additional natural sources of high‐activity PPO enzymes. While natural PPOs are plentiful, considerable discrepancies in enzyme properties and substrate specificities have been observed among PPOs obtained from various organisms (Yew et al. 2024). Conventional techniques for assessing enzyme activity tend to be inefficient, expensive, and limited in their applicability (Yan et al. 2024). Recently, bioinformatics strategies, which include homology modeling and molecular docking, in conjunction with high‐throughput screening methods, enable the swift and effective detection of optimal mutants that display favorable characteristics such as high enzyme activity, enantioselectivity, stability, and substrate specificity (Vasina et al. 2022). A compelling example of this is the work conducted by Zhou et al. (2017), who successfully identified two highly active polyphenol oxidases (PPOs) extracted from pear and sweet potato among 529 plant‐derived PPOs. They utilized bioinformatics techniques, including homology modeling and molecular docking. Comparisons of theaflavin conversion activities revealed that the PPOs sourced from Chinese pear and sweet potato outperformed the PPO from tea. This finding demonstrates that employing bioinformatics not only streamlines but also significantly enhances the overall efficiency and range of the enzyme screening process.

Although recombinant gene expression methodology is viewed as some of the most promising strategies for the large‐scale synthesis of polyphenol oxidase (PPO), its application is presently confined to laboratory‐scale (Beygmoradi et al. 2023). This constraint predominantly stems from the tendency of recombinant proteins to form inactive inclusion bodies (IBs). The problem of inactive IBs in *E. coli* expression systems could be addressed by in vitro refolding technology, which modifies the conditions in vitro to promote the refolding of expressed IBs into functional proteins (Bhatwa et al. 2021). Minimizing intermolecular interactions among the folding intermediates or partially folded species is crucial for improving both the quality and quantity of refolded proteins. Four essential factors affect the efficiency of protein refolding: the selection of solubilization agents, the protein concentration during the refolding process, the conditions of the refolding buffer, and the refolding method utilized (Singhvi et al. 2020). Singh et al. (2017) achieved successful refolding of recombinant *Camellia sinensis* polyphenol oxidase (CsPPO) by rapid dilution in refolding buffers containing 100 mM KPO_4_, 20 mM Tris, and 20 μM CuSO_4_, adjusted to pH 7.5. When the IBs were refolded in a refolding buffer containing 500 mM L‐arginine, the optimum PPO activity could reach 142.8 U/mg of protein, indicating a 247% increase in PPO activity compared with the refolding buffer without L‐arginine.

Protein engineering methods are increasingly being employed to enhance the structural and functional properties of PPO (Sodhi et al. 2024). Techniques such as directed evolution and protein structure‐guided engineering have proven effective in improving catalytic characteristics, including enzyme specificity, stability, and redox potential for various applications (Zhou et al. 2024). Both directed evolution and rational design utilize strategies like random mutagenesis and site‐directed mutagenesis, respectively, to introduce modifications that enhance the overall functionality of the enzyme (Bergeson and Alper 2024). The catalytic activity of tyrosinases derived from *Bacillus megaterium* (Bmtyrc) has been significantly enhanced through the implementation of a directed evolution strategy (Zhou et al. 2022). By employing this innovative approach, researchers successfully generated a mutant variant of Bmtyrc characterized by four distinct amino acid substitutions: N205D, D166E, D167G, and F197W. This engineered enzyme exhibited an impressive five‐fold increase in the yield of TFDG when compared to the wild‐type version, achieving a remarkable space–time yield of 35.35 g L^−1^ day^−1^. To enhance the preference, catalytic activity, and thermostability of PPO derived from *Rosa chinensis* (RcPPO), semi‐rational design was employed to conduct site‐directed mutagenesis (Luo, Hou, and Hu 2024). When comparing the kinetic profiles of the combinatory mutants, the triple mutant RcPPO‐FKI exhibited a 2.67‐fold increase in activity compared with the wild‐type. The level of TF1 obtained from the catalytic synthesis using the RcPPO‐FKI mutant was elevated by 109.9 μM compared with that of RcPPO, and the yield of TF1 from the RcPPO‐FKI mutant reached 70.65%.

#### 5.2.3 Proposed Research Strategies for Nonenzymatic TFs Synthesis

Due to intrinsic issues such as environmental effects and selectivity, the current techniques for the nonenzymatic production of TFs are not yet appropriate for large‐scale industrial use. Nevertheless, they present several innovative concepts and approaches alongside enzymatic synthesis techniques. Specifically, the integration of highly active catalysts with mild oxidants could achieve the efficient and selective production of TFs.

The concept of artificial mimic enzymes proposed as an innovative catalyst idea in the last century (Robert and Meunier 2022). It developed mimic catalysts that replicate the active sites and mechanisms of native enzymes, based on examining the chemical characteristics of enzyme catalysis. Unlike native enzymes, mimic enzymes offered benefits such as straightforward synthesis, stable storage, adaptable structures, and catalytic activities (Zou et al. 2022). Recently, advancements in nanotechnology have led to the rise of nano‐enzymes, which possess both the catalytic properties of enzymes and the distinctive physical and chemical traits of nanomaterials (Wang et al. 2019). Among these, the metal–organic framework (MOF), formed by the self‐assembly of metal ions (or metal clusters) and organic ligands through coordination chemistry, has been successfully utilized as a catalyst (Cai et al. 2021); (Wang and Astruc 2019). It has demonstrated significant potential for development in biosensing technology, quantitative detection of important targets, and catalytic degradation of organic compounds (Wei et al. 2020; Cheng et al. 2022).

Studies have demonstrated that the PPO genetic sequence contains two copper‐binding checkpoints, with each copper ion linked to three histidine residues through coordination bonds, forming an active site characterized by a specific three‐dimensional structure (Type 3 copper bridged; Ma et al. 2020). By mimicking the active structure of natural enzymes, copper‐based organic frameworks have been confirmed to exhibit oxidase‐like activity (Shen et al. 2024). Our team utilized Cu‐MOF for the catalytic synthesis of TFs. Investigations revealed that Cu‐MOF exhibited higher catalytic efficiency than native PPO, while also offering lower costs, enhanced stability, and improved recyclability compared to its native PPO.

#### 5.2.4 Research Strategies for Cost‐Effective Catechin Raw Materials

Catechins serve as precursors in the synthesis of TFs, and their content and composition are closely linked to the rate and yield of theaflavin production. However, the source of catechins remains a frequently overlooked limiting factor for the industrial production of TFs, largely due to the scarcity of research in this domain. Current studies have established that the concentration and composition of catechins during liquid fermentation play a role as critical as that of enzymes in the synthesis of TFs. Notably, a high concentration of catechins consistently exhibits PPO activity, while their composition has a direct impact on the synthesis rate, yield, and overall composition of TFs. Consequently, it is imperative to identify suitable raw materials from fresh tea leaves for the enzymatic synthesis of TFs through variety selection and advancements in planting technologies.

Research has indicated a strong correlation between the catechin content in fresh tea leaves and the resultant synthesis of TFs, particularly highlighting that a higher concentration of catechins, especially ester catechins, contributes positively to this process. Notably, there exists considerable variability in both the total catechin content and the proportion of ester catechins across different tea varieties, with variations sometimes amounting to several times (Kottawa‐Arachchi et al. 2022). In response to this variability, numerous researchers have undertaken the task of identifying tea resources rich in ester catechin content. For instance, Lin et al. (2005) successfully selected eight varieties of tea that exhibited an average ester catechin content exceeding 19% and a remarkable proportion of ester catechins surpassing 80%, all derived from an extensive screening of 780 different tea resources. Additionally, it has been observed that the levels of ester catechins in young tea shoots harvested in the summer showed no significant discrepancies when compared to those harvested in the spring (Deka et al. 2021). Considering the cost of raw materials, it means that fresh tea leaves during the summer are more appropriate for the industrial production of TFs. Furthermore, a range of environmental conditions, including soil composition, temperature, humidity, altitude, as well as factors associated with the harvesting process, such as the quality grade of the fresh leaves, also contribute significantly to the overall catechin composition found in fresh tea leaves (Yang et al. 2023).

Simultaneously, fertilization, pH levels, light management, and various other cultivation techniques also significantly influenced the catechin composition in tea leaves. For instance, in the tea variety “Huang Yan,” the C content peaked at 3.34 mg/g when pH was set at 3.5. Meanwhile, the levels of EC and ECG did not exhibit notable changes with varying pH, while EGC and GCG reached their maximum concentrations of 52.82 mg/g and 4.10 mg/g, respectively, at pH 4.0. Furthermore, EGCG content was highest at pH 4.5, measuring at 57.44 mg/g (Yan 2024). Low potassium fertilization would enhance the accumulation of EGCG (Yang et al. 2024). Additionally, zinc treatment in tea plants could increase the levels of trihydroxylated catechins (TRICs), including EGCG, EGC, and GC, in leaves with transient co‐overexpression that were 1.44 times greater than those in the control group, while tea callus exhibited a 50.83% increase in transient co‐overexpression compared to wild types (Hu, Yang, and He 2024; Hu, Li, et al. 2024). The effect of UV‐B irradiation duration on foliar catechin accumulation was assessed in two tea cultivars (Xia et al. 2008). A low impact rate and brief exposure effectively stimulated the primary catechins, leading to an overall increase in total catechin levels. However, excessive irradiation suppressed the increase of Epigallocatechin gallate (EGCG) more rapidly than that of the other catechins under optimal conditions.

## 6 Conclusion

In this review, recent advancements regarding the physiological activities and synthesis methods of theaflavins (TFs) were comprehensively summarized. The results shown suggest that TFs demonstrated considerable physiological benefits, such as antioxidant, antiviral, antibacterial, and properties related to cancer prevention and treatment. Nonetheless, research concerning the physiological impacts of TFs has predominantly been centered on cellular and animal studies, with a notable lack of clinical trials or research involving human subjects. Exploring particular mechanisms of action, pharmacokinetic characteristics, biosafety, and optimal dosages will establish a scientific foundation for the advancement of TFs as pharmaceuticals and functional foods. The industrial production of TFs still faces numerous challenges, primarily related to low production efficiency and product purity. Enzymatic oxidation methods, which are more efficient and environmentally friendly than chemical oxidation methods, have emerged as the main approach for synthesizing TFs. Recently, there has been considerable advancement in the study of enzymatic oxidation techniques, especially regarding the screening of enzymes and the optimization of reaction parameters. Nevertheless, the presence of inefficient and unstable enzymes continues to present challenges in achieving high‐purity TF products. To address these challenges, recombinant enzyme technologies such as heterologous expression and directed evolution have been employed to enhance the catalytic activity and substrate selectivity of PPO, while immobilized enzyme technology has been utilized to improve enzyme catalytic stability, facilitating storage and repeated use. These research advancements provide the potential for obtaining PPO with high catalytic activity. Nanozymes, such as metal–organic frameworks (MOFs), not only demonstrate catalytic activity similar to polyphenol oxidases (PPOs) but also offer considerable advantages regarding preparation expenses, thermal resilience, and stability across different pH levels. These features are anticipated to improve the use of biomimetic catalysis in the industrial production of TFs. In the future, as research progresses and breakthroughs are made in the physiological functions and synthesis techniques of TFs, their significant potential for enhancing human health and addressing disease treatment will undoubtedly be fully harnessed and realized.

## Author Contributions

**Junyi Chang:** investigation (equal), validation (equal), writing – original draft (equal). **Zhimei Zhou:** funding acquisition (equal), project administration (equal), supervision (equal), validation (equal), writing – review and editing (equal). **Shaolong Du:** conceptualization (equal), data curation (equal), funding acquisition (equal), investigation (equal), methodology (equal), project administration (equal), resources (equal), software (equal), supervision (equal), writing – review and editing (equal).

## Conflicts of Interest

The authors declare no conflicts of interest.

## Data Availability

This study did not generate any new datasets. All data analyzed are from publicly available sources, as cited in the manuscript.
